# Acute changes of global and longitudinal right ventricular function: an exploratory analysis in patients undergoing open-chest mitral valve surgery, percutaneous mitral valve repair and off-pump coronary artery bypass grafting

**DOI:** 10.1186/s12947-020-00218-x

**Published:** 2020-08-12

**Authors:** Marius Keller, Tim Heller, Tobias Lang, Johannes Patzelt, Juergen Schreieck, Christian Schlensak, Peter Rosenberger, Harry Magunia

**Affiliations:** 1 Department of Anesthesiology and Intensive Care Medicine, University Hospital Tuebingen, Eberhard-Karls-University, Hoppe-Seyler-Strasse 3, 72076 Tuebingen, Germany; 2 Chair of Visual Computing, Department of Computer Science, Eberhard-Karls-University, Sand 14, 72076 Tuebingen, Germany; 3 Department of Cardiology, Angiology and Intensive Care Medicine, University Hospital, University Heart Center Luebeck, Ratzeburger Allee 160, 23538 Luebeck, Germany; 4 Department of Cardiology and Angiology, University Hospital Tuebingen, Eberhard-Karls-University, Otfried-Mueller-Strasse 10, 72076 Tuebingen, Germany; 5 Department of Thoracic and Cardiovascular Surgery, University Hospital Tuebingen, Eberhard-Karls-University, Hoppe-Seyler-Strasse 3, 72076 Tuebingen, Germany

**Keywords:** Transesophageal echocardiography - three-dimensional echocardiography - right ventricular function - mitral valve surgery

## Abstract

**Background:**

Right ventricular (RV) function is an important prognostic indicator. The acute effects of cardiac interventions or cardiac surgery on global and longitudinal RV function are not entirely understood. In this study, acute changes of RV function during mitral valve surgery (MVS), percutaneous mitral valve repair (PMVR) and off-pump coronary artery bypass surgery (OPCAB) were investigated employing 3D echocardiography.

**Methods:**

Twenty patients scheduled for MVS, 23 patients scheduled for PMVR and 25 patients scheduled for OPCAB were included retrospectively if patients had received 3D transesophageal echocardiography before and immediately after MVS, PMVR or OPCAB, respectively. RV global and longitudinal function was assessed using a 3D multiparameter set consisting of global right ventricular ejection fraction (RVEF), tricuspid annular plane systolic excursion (TAPSE), longitudinal contribution to RVEF (RVEF_long_) and free wall longitudinal strain (FWLS).

**Results:**

Longitudinal RV function was significantly depressed immediately after MVS, as reflected by all parameters (RVEF_long_: 20 ± 5% vs. 13 ± 6%, *p* <  0.001, TAPSE: 13.1 ± 5.1 mm vs. 11.0 ± 3.5 mm, *p* = 0.04 and FWLS: −20.1 ± 7.1% vs. -15.4 ± 5.1%, p <  0.001, respectively). The global RVEF was slightly impaired, but the difference did not reach significance (37 ± 13% vs. 32 ± 9%, *p* = 0.15). In the PMVR group, both global and longitudinal RV function parameters were unaltered, whereas the OPCAB group showed a slight reduction of RVEF_long_ only (18 ± 7% vs. 14 ± 5%, *p* <  0.01). RVEF_long_ yielded moderate case-to-case but good overall reproducibility.

**Conclusions:**

TAPSE, FWLS and RVEF_long_ reflect the depression of longitudinal compared to global RV function initially after MVS. PMVR alone had no impact, while OPCAB had a slight impact on longitudinal RV function. The prognostic implications of these phenomena remain unclear and require further investigation.

## Background

Right ventricular (RV) dysfunction and RV failure assessed by echocardiography are major prognostic indicators of patient mortality after cardiac surgery [1]. In recent years, longitudinal RV function – describing the isolated contribution of baso-apical shortening to pressure generation and volume ejection – has been identified as an essential contributor to RV function in various conditions [2–6]. To date, heterogeneous data regarding global and longitudinal RV function after adult cardiac surgery exist but the available data point to a change in the sense of maintaining global but decreasing longitudinal function [7–13]. Although two-dimensional (2D) echocardiography is widely used for these analyses, 3D echocardiography is advantageous considering the complex geometry and heterogeneous myocardial deformation patterns of the right ventricle when compared to cardiac magnetic resonance [14]. However, thorough characterizations of the acute changes in RV longitudinal contraction throughout cardiac surgery or interventions employing innovative 3D imaging techniques are sparse.

Therefore, the rationale of this study is the exploratory assessment of longitudinal and global RV function by modern echocardiographic techniques. It is hypothesized that RV longitudinal and global function undergo distinct but potentially different changes immediately after cardiac surgery or cardiac interventions. Patients undergoing the following three cardiac procedures were investigated to depict potential procedure- and patient-specific differences:

1. open-chest mitral valve surgery (MVS)
2. percutaneous mitral valve repair (PMVR)
3. off-pump coronary artery bypass surgery (OPCAB)

A thorough characterization of longitudinal RV function was achieved by a multiparameter set derived from intraoperative three-dimensional (3D) speckle-tracking transesophageal echocardiography (TEE). This included tricuspid annular plane systolic excursion (TAPSE), free wall longitudinal strain (FWLS) and longitudinal contribution to RV ejection fraction (RVEF_long_) as recently reported [15, 16].

## Methods

### Study design

The study was designed as a retrospective observational cohort study with echocardiographic data extracted from an imaging database and clinical data from clinical patient records.

### Patients

Patients scheduled for isolated MVS or OPCAB via sternotomy between 2014 and 2017 were primarily screened for study inclusion as the MVS group or OPCAB group, respectively. The local ethics committee approved the retrospective enrollment of these cohorts (IRB #350/2015R). All patients were aged 18 years or older and were only enrolled if complete 3D echocardiographic studies were acquired before sternotomy and after sternal closure and if they matched the criteria (see below).

Adult patients with a percutaneous mitral valve intervention suffering from functional mitral regurgitation undergoing PMVR between July 2015 and April 2016 were included as the PMVR group. As these patients were enrolled prospectively, echocardiography was performed ultimately using a protocol involving 3D TEE studies before and after mitral valve clipping [17]. The local ethics committee approved the study (IRB #260/2015R) and informed consent was given prior to enrollment.

The present study was carried out in accordance with the Declaration of Helsinki.

### Cardiopulmonary bypass, cardioplegia and surgical technique (MVS group)

Conventional mitral valve repair or replacement was performed via median sternotomy, bicaval cannulation and ascending aorta cannulation for normothermic cardiopulmonary bypass. In all MVS patients, cold blood cardioplegia (Buckberg technique) was used. Patients received mitral valve replacement (bovine pericardial valves) or tailored mitral valve repair including implantation of an annuloplasty ring.

### Off-pump coronary artery bypass surgery

OPCAB was performed via sternotomy and pericardiotomy. According to the degree of coronary artery disease, up to five bypass grafts derived from both internal thoracic arteries or the left internal thoracic artery and a radial artery were used for total arterial revascularization.

### Percutaneous mitral valve repair

PMVR was performed as recently described [17, 18]. The procedure was carried out under either local anesthesia and sedation or general anesthesia.

### Anesthesia management

Anesthesia for MVS and OPCAB was provided according to an institutional standard protocol by experienced cardiac anesthesiologists as described recently [15, 19]. Induction was achieved intravenously by the administration of midazolam (0.1–0.15 mg / kg bw), sufentanil (0.3–0.5 μg / kg bw) and rocuronium (0.5–1 mg / kg bw). After endotracheal intubation, anesthesia was maintained using sevoflurane and continuous administration of sufentanil (0.8 to 1 μg / kg bw / h). Partial arterial CO_2_ pressures were kept between 35 and 40 mmHg and SpO_2_ was maintained above 95%. Positive end-expiratory pressure values ranged between 3 and 6 cmH_2_O. A mean artery pressure (MAP) of > 65 mmHg was maintained using titrated continuous norepinephrine infusion. Additional sympathomimetics – such as dobutamine, epinephrine or milrinone – were only used if the administration of NE alone was insufficient to establish hemodynamic stability after separation from cardiopulmonary bypass (CPB) or during OPCAB. For PMVR sedation, a continuous propofol infusion and intermittent piritramide bolus administration was used.

### Intraoperative echocardiography

TEE in the MVS and OPCAB group was performed at two time points: 1) after anesthesia induction but before sternotomy and 2) after (CPB and) chest closure. Hemodynamic stability was maintained for at least 5 min before image acquisition. TEE in the PMVR group was performed immediately before and after the application of the mitral valve clips. The institutional echocardiographic protocol has been reported previously [15, 20]. Briefly, standardized studies were acquired by specially trained investigators using a commercially available 3D probe (Philips X7-2t Matrix, Philips Healthcare Inc., Andover, MA, USA) in accordance with current recommendations [21, 22]. A representative 3D loop of the RV at a frame rate above 20 fps was achieved via the multi-beat acquisition of 4 heartbeats and a full projection of the right heart.

### Analysis of right ventricular global and longitudinal function

**Fig. 1:**
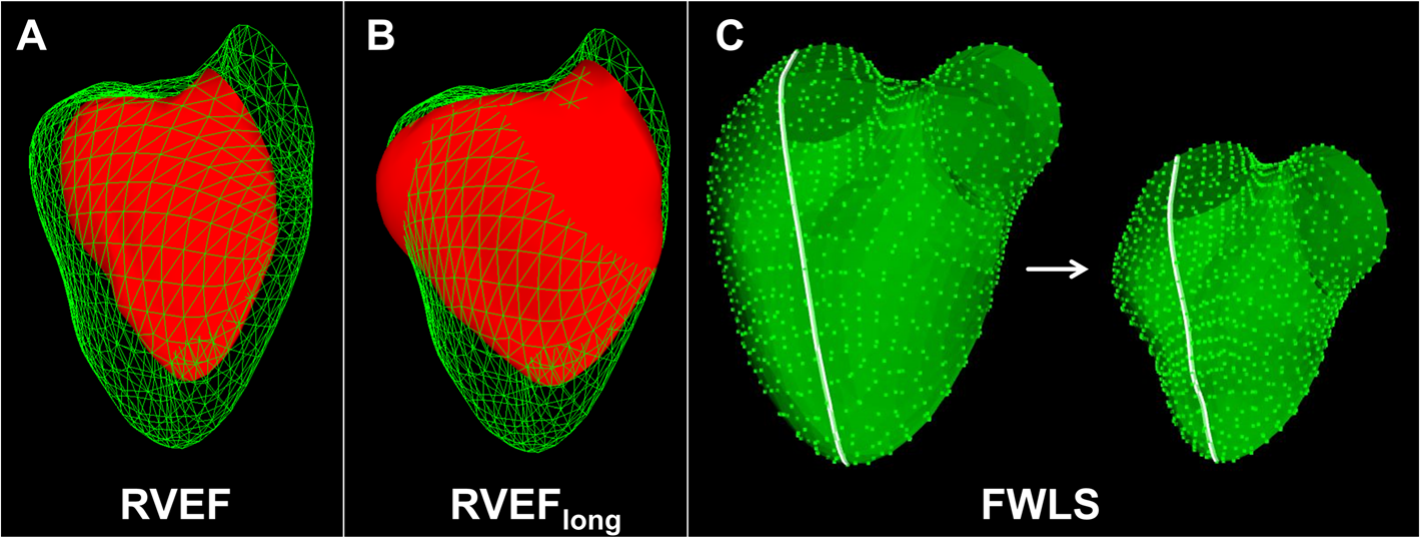
Assessment of global and longitudinal right ventricular function. Schematic illustration of right ventricular ejection fraction (RVEF, **a**), longitudinal contribution to right ventricular ejection fraction (RVEF_long_, **b**) and free wall longitudinal strain (FWLS, **c**) analysis employing meshes from the same patient derived from three-dimensional speckle-tracking transesophageal echocardiography

RV longitudinal function was assessed employing meshes derived from 3D speckle-tracking echocardiography (STE). As reported previously, a commercially available STE volume analysis package (4D RV-Function 2.0, Tomtec Imaging Systems GmbH, Unterschleissheim, Germany) was used to assess TAPSE and global 3D RVEF (Fig. 1a) while creating endocardial mesh models of the cardiac cycle with the same process [15]. The meshes were then postprocessed to measure peak systolic 3D right ventricular free wall longitudinal strain on the mesh surface (FWLS, Fig. 1c) using a recently published software solution [15]. In brief, FWLS reflects the maximum shortening of the endocardial contour from the lateral tricuspid annulus to the RV apex in three-dimensional space and is given with a percentage. Additionally, the mesh models were imported into the ReVISION software for the analysis of the longitudinal contribution to RVEF (RVEF_long_, Fig. 1b) as reported by Lakatos et al. [16]. This tool estimates the ejection fraction of the RV if only long-axis shortening is responsible for systolic volume output.

### Reproducibility and reliability of RVEF_long_

TAPSE is a well-established clinical parameter and the excellent reproducibility and reliability of FWLS have been demonstrated recently [15]. Since the intraobserver and interobserver variabilities of RVEF_long_ analysis by TEE had not been assessed in our institution, blinded double measurements of pre- and postinterventional datasets of 20 randomly selected patients were performed. For intraobserver analysis the measurements were performed by the same investigator (TH), while a second investigator (MK) analyzed RVEF_long_ in those patients for the assessment of interobserver variability.

### Statistical analysis

If samples were normally distributed according to the D’Agostino-Pearson test, the mean values ± standard deviations were used to describe the sample. For the comparison of patient characteristics between the three study groups, one-way ANOVA with Welch’s correction (mean values) and Chi squared test (proportions) were used. The differences in RV longitudinal function parameters before and after the procedures were compared using paired Student’s t-tests with Welch’s correction. For the comparison of vasopressor unpaired Student’s t-tests were used. The *p*-values were considered statistically significant as follows: moderate for *p* <  0.05 (*), medium for *p* <  0.01 (**), and high for *p* <  0.001 (***). For intra- and interobserver variability analyses, Pearson’s correlation coefficients were calculated in the case of normal distribution; otherwise, Spearman’s coefficients were used. Additionally, linear regression, intraclass correlation and Bland-Altman analysis were performed to compare repeated measurements. The Bland-Altman limits of agreement (LOA) were defined as the interval of bias ±1.96-fold standard deviation containing 95% of the measurements.

Excel 2010 (Microsoft Corp., Redmond, WA, USA) and Prism 8 (GraphPad Software, Inc., La Jolla, CA, USA) were used for data documentation and statistical analysis. The presentation of data was performed in accordance with the STROBE statement [13].

## Results

### Baseline and peri-procedural characteristics

Twenty MVS patients, 23 PMVR patients and 25 OPCAB patients were included. The characteristics of the study groups resemble typical cohorts for each procedure and are summarized in Table 1. PMVR yielded a reduction of mitral regurgitation in all patients. OPCAB yielded successful revascularization in all patients. Notably, 16 out of the 25 patients received right coronary revascularization. The dosages of vasopressors and inotropics used in the MVS and OPCAB patients are listed in Table 2. Compared to the baseline conditions, significantly higher levels of inotropic support were administered by continuous milrinone and dobutamine infusions at the end of MVS. OPCAB patients however only received significantly higher dosages of norepinephrine at the end of surgery. Patients undergoing PMVR did not receive vasopressors or inotropics. None of the patients in the OPCAB or PMVR group died during in-hospital follow-up. MVS was successful in all patients, yet one patient died due to hemorrhagic complications after veno-venous extracorporeal membrane oxygenation for the treatment of lung failure.

**Table 1:** Characteristics of the study groups

	MVS group (***n*** = 20)	PMVR group (***n*** = 23)	OPCAB group (***n*** = 25)	***p***-value
**Male, n**	13 (65%)	13 (57%)	19 (76%)	0.21
**Age, years**	60 ± 20	74 ± 12	71 ± 9	0.03*
**NYHA class**	II (I, III)	III (III, VI)	II (I, III)	< 0.001***
**EuroSCORE II, %**	4.0 ± 7.2	13.5 ± 12.4	6.0 ± 9.4	< 0.01**
**Type of surgery**				
Replacement	7 (35%)	–	–	
Repair	13 (65%)	–	–	
**General anesthesia**	20 (100%)	5 (22%)	25 (100%)	< 0.001***
**Nr. of clips applied, n**				
1	–	12 (52%)	–	
2	–	10 (44%)	–	
3	–	1 (4%)	–	
**reduced LVEF, n**	2 (10%)	21 (91%)	13 (52%)	< 0.001***
**elevated PAP**_**sys**_**, n**	5 (25%)	16 (70%)	2 (8%)	< 0.001***
**CAD, n**	0 (0%)	19 (89%)	25 (100%)	< 0.001***
**RCA revascularization, n**	–	–	16 (64%)	
**RVEDV, ml**	137 ± 37	168 ± 76	145 ± 49	0.24
**RVESV, ml**	91 ± 38	113 ± 58	89 ± 43	0.25
**GFR < 50 ml/min, n**	2 (10%)	7 (30%)	5 (20%)	0.25
**CPB / procedure time, min**	134 ± 32	106 ± 49	227 ± 53	< 0.001***

*CPB* cardiopulmonary bypass, *CHD* coronary artery disease, *GFR* glomerular filtration rate, *LVEF* left ventricular ejection fraction (considered reduced if < 50% preoperatively), *NYHA* New York Heart Association, *PAP*_*sys*_ systolic pulmonary artery pressure (considered increased if > 30 mmHg preoperatively), *RCA* right coronary artery, *RVEDV* right ventricular end-diastolic volume (measured intraoperatively before sternotomy / before mitral valve clipping), *RVESV* right ventricular end-systolic volume (measured intraoperatively before sternotomy / before mitral valve clipping)

**Table 2:** Dosages of vasopressors and inotropics during image acquisition in the cardiac surgery groups

	Pre	Post	***p***-value
**MVS group**			
**Norepinephrine,** μg**/kg/min**	0.06 ± 0.07	0.07 ± 0.06	0.19
**Milrinone, μg/kg/min**	0 ± 0	0.25 ± 0.21	< 0.001***
**Dobutamine,** μg**/kg/min**	0 ± 0	2.17 ± 2.77	< 0.001***
**Epinephrine, μg/kg/min**	0 ± 0	0.01 ± 0.02	0.31
**Vasopressin, IU/h**	0 ± 0	0 ± 0	1.0
**OPCAB group**			
**Norepinephrine, μg/kg/min**	0.06 ± 0.05	0.14 ± 0.08	< 0.001***
**Milrinone, μg/kg/min**	0.05 ± 0.11	0.05 ± 0.10	1.0
**Dobutamine, μg/kg/min**	0.20 ± 1.00	0.07 ± 1.70	0.17
**Epinephrine, μg/kg/min**	0 ± 0	0 ± 0	0.33
**Vasopressin, IU/h**	0 ± 0	0 ± 0	1.0

### Right ventricular global and longitudinal function

**Fig. 2:**
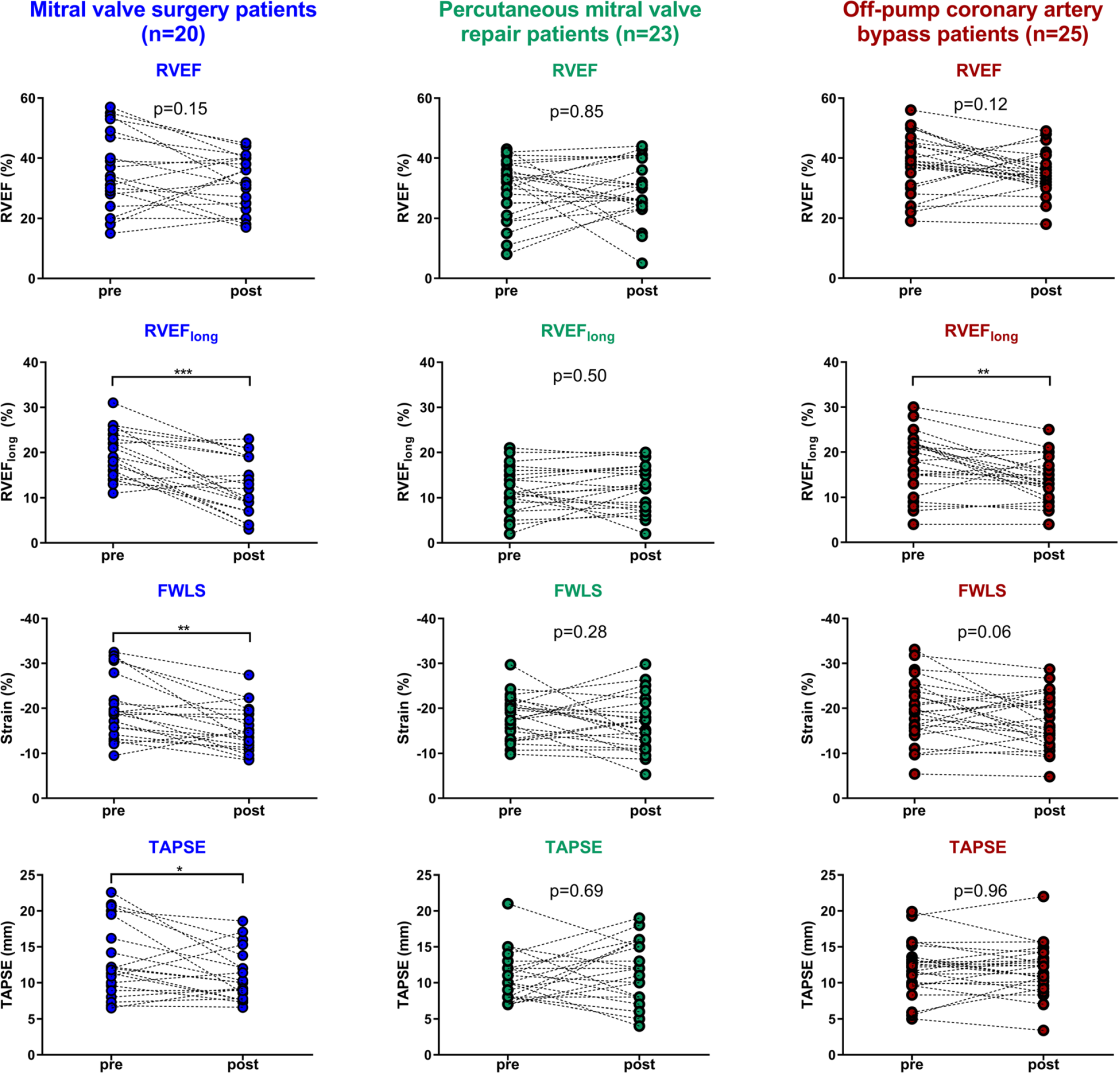
Results of gobal and longitudinal right ventricular function analysis. Global and longitudinal right ventricular function of mitral valve surgery patients, percutaneous mitral valve repair patients and off-pump coronary artery bypass patients initially before (pre) and after (post) the procedure. FWLS = free wall longitudinal strain, RVEF = right ventricular ejection fraction, RVEF_long_ = longitudinal contribution to right ventricular ejection fraction, TAPSE = tricuspid annular plane systolic excursion. * = *p* < 0.05, ** = *p* < 0.01, ** = *p* < 0.01, *** = *p* < 0.001 (paired Student’s t-test)

The echocardiographic datasets of all included patients were regarded as suitable for image analysis. The results of RV global and longitudinal function analyses by TAPSE, FWLS and RVEF_long_ are displayed in Fig. 2. The numeric values are given in Table 3. Compared to the MVS and OPCAB patients, patients in the PMVR group showed decreased RVEF values before clipping that did not deteriorate significantly during the procedure. The MVS patients had significantly decreased longitudinal RV function initially after weaning from CPB reflected by all employed measures (TAPSE, RVEF_long_, FWLS) compared to baseline values. Accordingly, with a relative reduction of 35% of the means (abs. -7.2%, CI: − 9.8 to − 4.5%) RVEF_long_ was more severely reduced than FWLS (relative reduction 23%, abs. 4.7%, CI: 2.0 to 7.5%) or TAPSE (relative reduction 15%, abs. -2.1 mm, CI − 4.1 to − 0.1 mm) in the MVS group. In the OPCAB group, only RVEF_long_ was significantly reduced immediately after revascularization.

**Table 3:** Numeric results of global and longitudinal right ventricular function analysis

	MVS group (***n*** = 20)			PMVR group (***n*** = 23)			OPCAB group (***n*** = 25)		
	pre	post	***p***-value	pre	post	***p***-value	pre	post	***p***-value
**RVEF, %**	37 ± 13	32 ± 9	0.15	30 ± 10	29 ± 10	0.85	38 ± 10	35 ± 7	0.12
**TAPSE, mm**	13.1 ± 5.1	11.0 ± 3.5	0.04*	10.6 ± 3.4	11.0 ± 4.4	0.69	10.6 ± 3.4	11 ± 3.7	0.96
**FWLS, %**	− 20.1 ± 7.1	−15.4 ± 5.1	< 0.001***	−17.9 ± 4.9	−16.4 ± 6.2	0.28	− 19.6 ± 6.9	- 17.2 ± 6.0	0.06
**RVEF**_**long**_**, %**	20 ± 5	13 ± 6	< 0.001***	12 ± 5	12 ± 5	0.50	18 ± 7	14 ± 5	< 0.01**

*FWLS* free wall longitudinal strain, *RVEF* right ventricular ejection fraction, *RVEF*_*long*_ longitudinal contribution to right ventricular ejection fraction, *TAPSE* tricuspid annular plane systolic excursion

### Observer variability of RVEF_long_

**Fig. 3:**
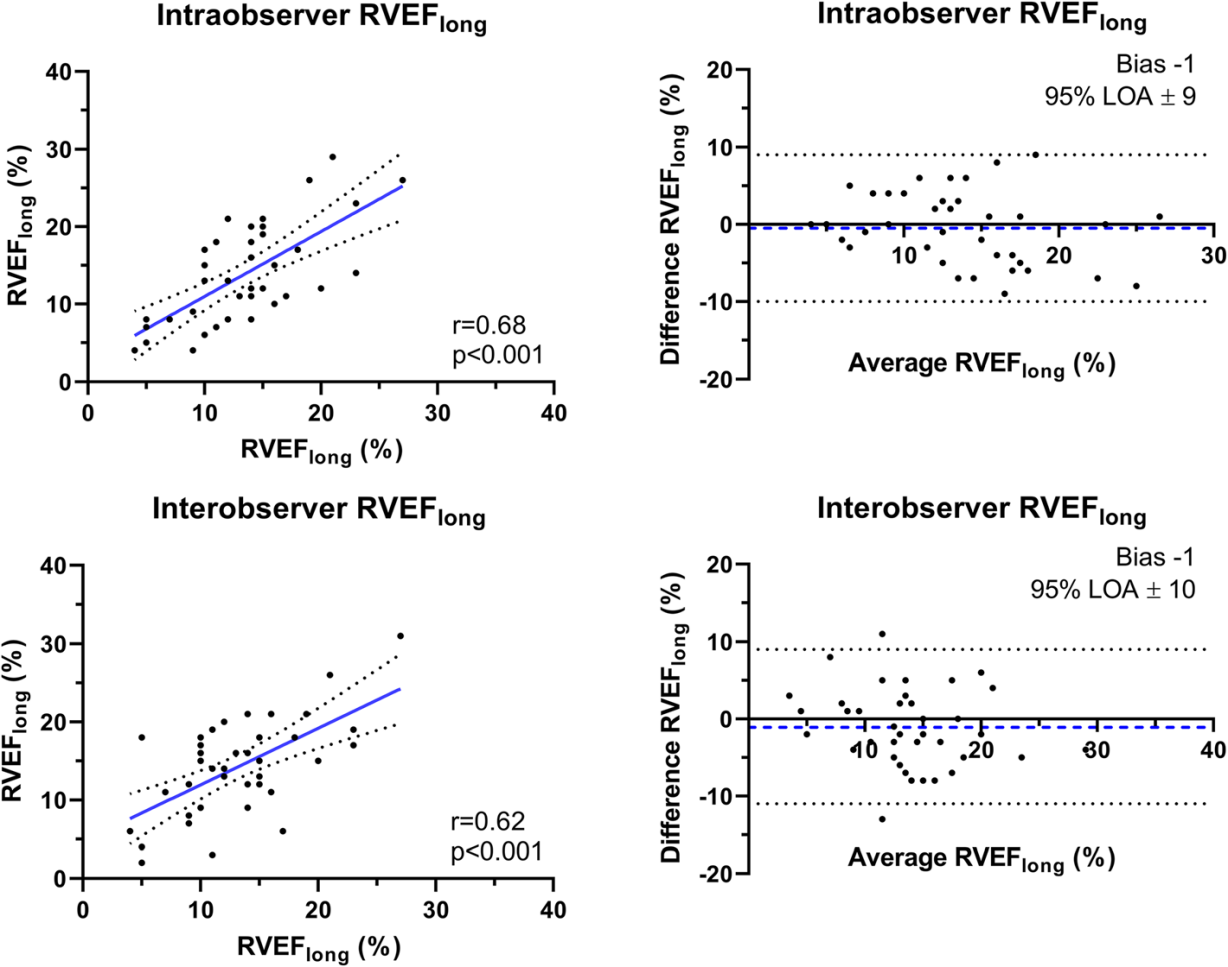
Reproducibility of RVEF_long_. Reproducibility of measurements of longitudinal contribution to right ventricular ejection fraction (RVEF_long_). Intraobserver (top) and interobserver analyses (bottom) by linear regression (left) and Bland-Altman plots (right). Left: Linear regression lines (blue) with dotted 95% confidence slopes, right: bias (blue dashed lines) and 95% limits of agreement (LOA, straight dotted lines), 95%LOA = 95% limits of agreement, r = Pearson’s correlation coefficient

Linear regression, correlation coefficients and Bland-Altman plots of intra- and interobserver analyses of RVEF_long_ measurements are displayed in Fig. 3. Intraclass correlation coefficients were 0.800 and 0.754 for intra- and interobserver analyses, respectively. The correlation coefficients and 95% LOA revealed moderate agreement between repeated measurements. However, average values reflected by low biases showed good overall comparability.

## Discussion

**Fig. 4:**
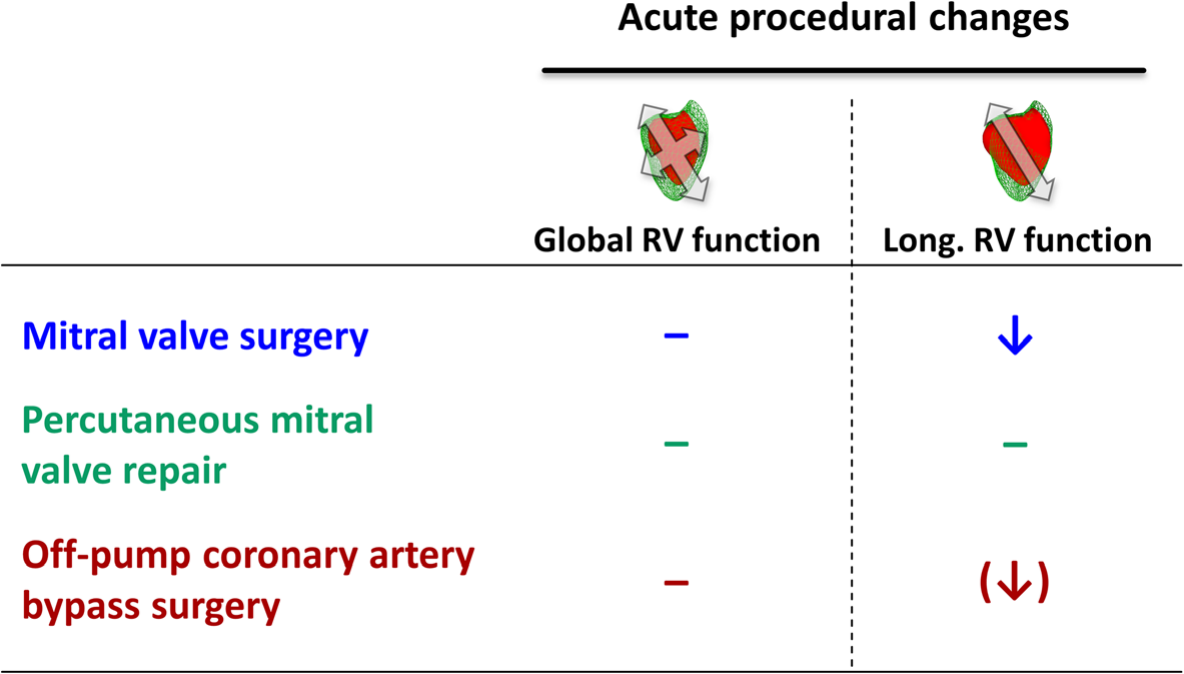
Summary of the study results. Graphical overview of the acute procedural changes of global and longitudinal right ventricular (RV) function in each study cohort

To our knowledge, this is the first study to investigate right ventricular longitudinal function initially before and immediately after open-chest mitral valve surgery, percutaneous mitral valve repair and off-pump coronary artery bypass grafting employing modern 3D TEE-derived parameters. As hypothesized, longitudinal RV function showed distinct procedure- or patient-specific alterations. All employed parameters of longitudinal RV function were significantly reduced after MVS compared to baseline values. The deterioration of global RVEF was less pronounced and did not reach statistical significance. It is conceivable however, that this difference turns out statistically different in a larger study cohort. Patients undergoing isolated treatment of mitral valve regurgitation via PMVR did not show a systematic alteration of global or longitudinal RV function. In a group of patients undergoing off-pump coronary artery bypass surgery, only RVEF_long_ was significantly reduced at the end of surgery. A graphical summary of the study results is displayed in Fig. 4.

The reproducibility of RVEF_long_ was limited when comparing repeated measurements of the same echocardiographic images. However, Bland-Altman analysis revealed good overall comparability of group averages with low biases (− 1% and − 1% for intra- and interobserver analysis, respectively). In a recent study, we demonstrated the excellent reproducibility of FWLS regarding both case-to-case and overall comparability, reflecting a potential advantage of FWLS analysis compared to RVEF_long_ during clinical implementation [15].

Over the last few decades, there has been substantial debate regarding RV contraction patterns and their alterations during RV stress [23]. In the setting of cardiac surgery, various studies have focused on the adaptive mechanisms regarding RV contraction after sternotomy, pericardiotomy, cardioplegia and cardiopulmonary bypass [7, 10–12]. Intraoperative TEE is known to have the potential to have a major impact on surgical decision-making [24]. Considering the regimen of postoperative respiratory care, depressed RV longitudinal function might contribute to decisions of administering prolonged inotropic support, potentially affecting outcomes [25]. However, the analysis of established and innovative measures of RV longitudinal function – such as TAPSE, longitudinal strain or RVEF_long_ – might distort the fact that RV function is altered morphologically but not effectively in the overall patient population undergoing cardiac surgery [26].

While long-term alterations in longitudinal RV function are increasingly being understood [27], the functional changes immediately after MVS employing cardiopulmonary bypass have not been investigated thoroughly. As a decrease in longitudinal RV function after MVS has been demonstrated [12], our results underline those changes as early as during the course of surgery. This phenomenon should be taken into consideration when RV failure appears to be clinically or hemodynamically unlikely, as longitudinal dysfunction might be compensated by increased circumferential shortening without prognostic implications [13]. In this setting, echocardiographic measures of global RV function – such as 3D-derived RVEF – or invasive hemodynamic data might be preferable [28].

A study of major importance characterizing RV contraction patterns in patients undergoing elective coronary artery bypass grafting (CABG) was recently published by Donauer et al. [8]. While right ventricular systolic function remained unaltered, RV longitudinal contraction reflected by 3D-derived strain analysis was decreased, which is in line with our findings in the MVS group. Interestingly, pericardiotomy alone did not alter short-term longitudinal or circumferential RV strain which might explain the preservation of FWLS in our OPCAB patients. Keyl et al. conducted a similar study investigating patients undergoing either surgical aortic valve repair or transcatheter aortic valve implantation [7]. Compared to pre-interventional values, longitudinal but not global RV function was reduced after surgical but not percutaneous intervention. We observed similar effects when comparing surgical (MVS) to percutaneous (PMVR) valve repair patients. In this context, surgical manipulation of the mitral valve and its annulus as well as CPB might have a larger impact on longitudinal RV function than solely clipping the leaflets. The comparison of MVS and PMVR patients is yet hindered by the far worse baseline RV function in our patients suffering from functional mitral regurgitation.

In contrast to the MVS group, our OPCAB patients showed no reduction of global but a slight decrease in longitudinal RV function (only RVEF_long_). This finding might point to a strength of RVEF_long_ compared to TAPSE or FWLS: As a volumetric parameter RVEF_long_ reflects longitudinal function of the whole RV and might thus be more sensitive to detect reductions. TAPSE predominantly includes the lateral tricuspid annulus and FWLS analysis only includes the RV free wall.

The possible reasons for a reduction in longitudinal RV function after cardiac surgery are manifold. Pericardiotomy – as mentioned above – has been frequently discussed to be a major contributor to reduced longitudinal RV shortening [29]. However, altered contraction after pericardial incision and closure appears to have long-term effects due to a switch in RV geometry rather than initially affecting longitudinal fiber shortening directly [10]. Decreased coronary blood flow – possibly induced by pericardiotomy or cardioplegia – usually leads to transient inner myocardial wall ischemia [30]. As the subendocardial fibers of the right ventricle are orientated longitudinally, this phenomenon potentially accounts for a resulting reduction predominantly of longitudinal function [23]. As post-CPB inflammatory effects leading to transient hypodynamic or vasoplegic conditions are frequent, patients regularly require increased vasopressor and inotropic support after weaning from CPB to maintain an adequate MAP [31]. Increased levels of inotropics might also contribute to altered RV contraction patterns. Dobutamine for instance was recently described to increase RVEF without significantly increasing TAPSE in patients with biventricular heart failure [32].

In general, the reasons for reduced longitudinal RV function due to open-chest mitral valve surgery are of great academic interest and further research is necessary to understand the underlying mechanisms. In the clinical setting however, it is potentially more valuable to simply accept this phenotype of myocardial deformation and rely on the likelihood of its limited prognostic implications during an uneventful surgical course [7]. Possible implications of this phenomenon are altered diagnostic algorithms to assess RV function after cardiac surgery, as discussed in prior works [28]. In fact, interdisciplinary decisions based on integrated approaches should essentially involve all available measures of RV function to provide optimal patient care before, during and after cardiac procedures.

### Limitations

There are a few limitations of our report. The sample sizes for the study groups were small, whereby statements regarding prognosis or universal applicability of the observed effects are limited. The compilation of the study groups comprised a comparison of two different etiologies of mitral valve pathologies, namely degenerative and functional mitral regurgitation, as well as patients undergoing revascularization for coronary artery disease. Furthermore, patients undergoing general anesthesia (MVS, OPCAB) were compared to sedated patients (majority of PMVR), where the type of anesthesia potentially acts as a confounder. Substantial differences in inotropic and vasopressor support between the three groups might further influence RV contraction. One of the substantial differences between the study groups was the significantly different number of patients with elevated pulmonary artery pressures, which potentially influences data interpretation. Moreover, no invasive hemodynamic data were available to compare them to the echocardiographic values, as pulmonary artery catheters are not routinely employed during cardiac surgery or PMVR at our institution. Data from other imaging modalities such as cardiac magnetic resonance or transthoracic echocardiography – especially from pre- or postoperative time points – were not systematically available.

## Conclusion

Longitudinal right ventricular systolic function assessed by modern three-dimensional echocardiography is more severely decreased than global right ventricular function immediately after mitral valve surgery. The longitudinal contribution to right ventricular ejection fraction (RVEF_long_) was more reduced than TAPSE or FWLS in MVS patients but showed limited case-to-case reproducibility. The acute effects of off-pump coronary artery bypass surgery on RV function comprise of a slight reduction of longitudinal function reflected only by RVEF_long_ analysis. In comparison, patients undergoing percutaneous mitral valve repair show decreased global and longitudinal right ventricular function at baseline that show no further deterioration after the procedure. Further research is necessary to unveil the possible prognostic implications associated with these phenomena.

## Data Availability

The datasets used and/or analyzed during the current study are available from the corresponding author on reasonable request.

## Abbreviations

2D: two-dimensional
3D: three-dimensional
abs: absolute
bw: body weight
CABG: coronary artery bypass grafting (surgery)
CAD: coronary artery disease
CI: confidence interval
CPB: cardiopulmonary bypass
fps: frames per second
FWLS: free wall longitudinal strain
GFR: glomerular filtration rate
LOA: limits of agreement
LVEF: left ventricular ejection fraction
MAP: mean artery pressure
MICS: minimally invasive cardiac surgery
MVS: mitral valve surgery
NYHA: New York Heart Association
OPCAB: off-pump coronary artery bypass (surgery)
PAP_sys_: systolic pulmonary artery pressure
PMVR: percutaneous mitral valve repair
RCA: right coronary artery
RV: right ventricular / right ventricle
RVEDV: right ventricular end-diastolic volume
RVEF: right ventricular ejection fraction
RVEF_long_: longitudinal contribution to right ventricular ejection fraction
RVESV: right ventricular end-systolic volume
STE: speckle-tracking echocardiography
TAPSE: tricuspid annular plane systolic excursion
TEE: transesophageal echocardiography
